# Analysis of endothelial progenitor cell subtypes as clinical biomarkers for elderly patients with ischaemic stroke

**DOI:** 10.1038/s41598-023-48907-7

**Published:** 2023-12-09

**Authors:** Rais Reskiawan A. Kadir, Kamini Rakkar, Othman A. Othman, Nikola Sprigg, Philip M. Bath, Ulvi Bayraktutan

**Affiliations:** 1 Academic Unit of Mental Health and Clinical Neuroscience, The University of Nottingham, Nottingham, UK; 2 Academic Unit of Mental Health and Clinical Neuroscience, Queens Medical Centre, School of Medicine, University of Nottingham, Derby Road, Nottingham, NG7 2UH UK

**Keywords:** Adult stem cells, Regeneration, Neurology

## Abstract

Endothelial progenitor cells (EPCs), expressing markers for stemness (CD34), immaturity (CD133) and endothelial maturity (KDR), may determine the extent of post-stroke vascular repair. Given the prevalence of stroke in elderly, this study explored whether variations in plasmatic availability of certain EPC subtypes could predict the severity and outcome of disease in older patients. Blood samples were collected from eighty-one consented patients (≥ 65 years) at admission and days 7, 30 and 90 post-stroke. EPCs were counted with flow cytometry. Stroke severity and outcome were assessed using the National Institutes of Health Stroke Scale, Barthel Index and modified Rankin Scale. The levels of key elements known to affect EPC characteristics were measured by ELISA. Diminished total antioxidant capacity and CD34 + KDR + and CD133 + KDR + counts in early phases of stroke were associated with disease severity and worse functional outcome at day 90 post-stroke. Baseline levels of angiogenic agent PDGF-BB, but not VEGF, positively correlated with CD34 + KDR + numbers at day 90. Baseline LDL-cholesterol levels were inversely correlated with CD34 + KDR+, CD133 + KDR + and CD34 + CD133 + KDR + numbers at day 90. Close correlation between baseline CD34 + KDR + and CD133 + KDR + counts and the outcome of stroke proposes these particular EPC subtypes as potential prognostic markers for ischaemic stroke.

## Introduction

Ischaemic stroke (IS) develops through an interference with blood supply to the brain and continues to be one of the leading causes of mortality and morbidity in the world^1^. The prevalence of stroke doubles every 10 years after the age of 55 and about two-third of all stroke patients are older than 65 years^1,2^. Although various mechanisms can initiate and exacerbate the development of atherosclerotic disease throughout chronological ageing, endothelial dysfunction (ED), accompanied by impaired vascular relaxation and inflammation, is widely considered as a key pathology behind the structural and functional alterations in the vascular system^3^.

The endothelium is important in preserving vascular homeostasis^4,5^. Endothelial progenitor cells (EPCs), released from bone marrow, play an instrumental role in maintaining appropriate endothelial function by re-endothelialisation of cerebral blood vessels after IS^6,7^. Similar to embryonic angioblasts, EPCs are equipped with an inherent capacity to proliferate, migrate and differentiate. Hence, non-haematopoietic cells (CD45-) expressing a variety of markers for stemness (CD34+), immaturity (CD133+) and endothelial cell maturity (KDR+) are regarded as distinct EPC subtypes in circulation^7,8^. Circulating EPCs can also negate the deleterious effects of cerebral ischaemia by inducing angiogenesis and vasculogenesis through secretion of various trophic factors^9^. However, the mobilisation, survival and the reparative capacity of EPCs are negatively affected by chronological ageing in that age-related changes in the availability of circulatory cytokines, chemokines and growth factors, appear to play an instrumental role^10^. Besides, advancing age renders EPCs susceptible to internal changes and environmental factors such as oxidative stress which, markedly influences the number of circulating EPCs and contributes to age-mediated gradual loss of endothelium in cerebrovasculature and to the pathogenesis of IS^11^.

Oxidative stress represents a common pathology during the acute phase of an IS and is characterised by the excessive availability of reactive oxygen species (ROS)^12^. Both the enhanced production and reduced metabolism of ROS may account for this phenomenon. Exposure to excessive levels of ROS irreversibly damages macromolecules, including DNA and proteins, exacerbates ED and may adversely affect EPC characteristics^13^.

In light of the above, we hypothesised that variations in numbers of different EPC subtypes might correlate with the severity and outcome of IS and therefore serve as prognostic markers for stroke. In addition, the study also investigated the correlation between the plasmatic profile of inflammatory cytokines, growth factors and angiogenic factors known to affect EPC characteristics and disease severity and outcome.

## Materials and methods

### Study participants

Data from eighty-one older (≥ 65 years old) IS patients recruited for The Dunhill Medical Trust EPC study (DMT EPC study, NCT02980354) between February 2017 and November 2019 were used for this substudy. IS was defined as a sudden focal neurological deficit persisting longer than 24 h with no evidence of cerebral haemorrhage on imaging. The DMT EPC study was a single-centre, prospective, observational, case-controlled study, performed in accordance with the ethical standards for human experimentation established by the Declaration of Helsinki. Details of the study have already been reported elsewhere^14^. The study protocol was reviewed and approved by West Midlands-Coventry & Warwickshire Research Ethics Committee (REC number: 16/WM/0304). Written informed consent was obtained from all participants for their anonymised information to be published in this study.

National Institutes of Health Stroke Scale (NIHSS), modified Rankin Scale (mRS) and Barthel Index (BI) were used to measure patients’ neurological and functional status on admission (baseline, BL) and days 7, 30 and 90 post-stroke. As all patients received guideline-based medical therapy and underwent an appropriate rehabilitation programme, patients’ EPC counts and neurological outcomes were unlikely to be differently affected by these treatments.

### Blood sampling and flow cytometry

Blood samples (~ 30 mL) were collected from participants at the abovementioned time points to cover acute (within the first 48 h of stroke, baseline, BL), subacute (day 7, D7) and chronic (days 30 and 90, D30 and D90, respectively) phases of stroke. Six mL of blood samples were used to count circulating EPC subtypes by flow cytometry where non-haematopoietic cells (CD45-) co-expressing two or more cell surface markers for stemness (CD34 +), immaturity (CD133 +) and endothelial maturity (KDR +), i.e., CD45-CD34 + CD133 +, CD45-CD34 + KDR +, CD45-CD133 + KDR + and CD45-CD133 + CD34 + KDR +, were counted. To determine the percentage of each EPC subpopulation 1 million total events were acquired. The researchers who carried out the experiments remained blinded to subject characteristics throughout the study to avoid bias.

### Biochemical profile assessment

The biochemical profile of patient plasma samples was scrutinised for major angiogenic promoters and inhibitors, namely VEGF (DVE00), PDGF-BB (DBB00), thrombospondin-1 (DTSP10), thrombospondin-2 (DTSP20), endostatin (Abcam, ab100508) and angiostatin (Abcam, ab99973) and inflammatory cytokines and chemokines, namely TNF-α (DTA00D), SDF-1 (DSA00), G-CSF (DCS50), using specific ELISAs. Changes in total antioxidant capacity (TAC), determined by the sum of endogenous and food-derived antioxidants, were also measured in plasma using a commercial kit, TAC assay kit (Abcam, ab65329). Unless otherwise stated, all kits mentioned were from R&D systems.

### Statistical analysis

The statistical analyses were performed using SPSS package version 26 (SPSS Inc., USA) and GraphPad Prism 8.0 statistical software package (GraphPad Software Inc). Spearman correlation analysis was performed to capture the association between all the variables. Spearman’s correlation coefficients (r) were utilised to summarise the relationship between all the variables. *p* < 0.05 was considered as significant.

### Ethics approval

The Dunhill Medical Trust EPC study protocol was reviewed and approved by West Midlands—Coventry & Warwickshire Research Ethics Committee (16/WM/0304).

## Results

### Study population

Blood samples were collected from a total of 81 subjects with lacunar (n = 38) or cortical (n = 43) stroke on the basis of clinical syndrome and neuroimaging of whom 69% were male (n = 56). The median age at the diagnosis was 76 years. More than half of the patients had a history of smoking (n = 48), consumed alcohol (n = 51) and were hypertensive (n = 49). Over a quarter of all patients had atrial fibrillation (AF, n = 23) and were hyperlipidaemic (n = 24). Over 15% of all patients also had a previous history of transient ischaemic attack (TIA, n = 15) and IS (n = 12) and were diabetic (n = 17). Hence, over 40% of patients were on statins (n = 38), ACE inhibitors (n = 34) and anti-platelets (n = 33). The use of calcium channel blockers (n = 17), beta blockers (n = 15) and glucose-lowering (n = 13) agents amongst patients was frequent (Table 1).

**Table 1:** Demographic and baseline clinical characteristics of the patients with ischaemic stroke.

	Patients *n* = 81
Age (years) median	76
Sex	
Male (*n*)	56
Type of stroke	
Lacunar (*n*)	43
Cortical (n)	38
History	
Smoking (*n*)	48
Alcohol (*n*)	51
Hypertension (*n*)	49
Diabetes (*n*)	17
AF (*n*)	23
Hyperlipidemia (*n*)	24
TIA (*n*)	15
IS (*n*)	12
ICH (*n*)	0
Medications	
Statins (*n*)	38
Ace inhibitor (*n*)	34
CCB (*n*)	17
Beta blocker (*n*)	15
Nitrates (*n*)	3
Anti-platelet (*n*)	33
Insulin (*n*)	2
Glucose lowering	13
Severity	
NIHSS on admission (mean)	4.85
Outcome day 90	
mRS ≤ 2 (*n*)	82.4%
BI ≥ 90 (*n*)	92.1%

*BI* Barthel index, *CD* cluster differentiation, *mRS* modified ranking score, *NIHSS* national institutes of health stroke scale.

**Figure 1:**
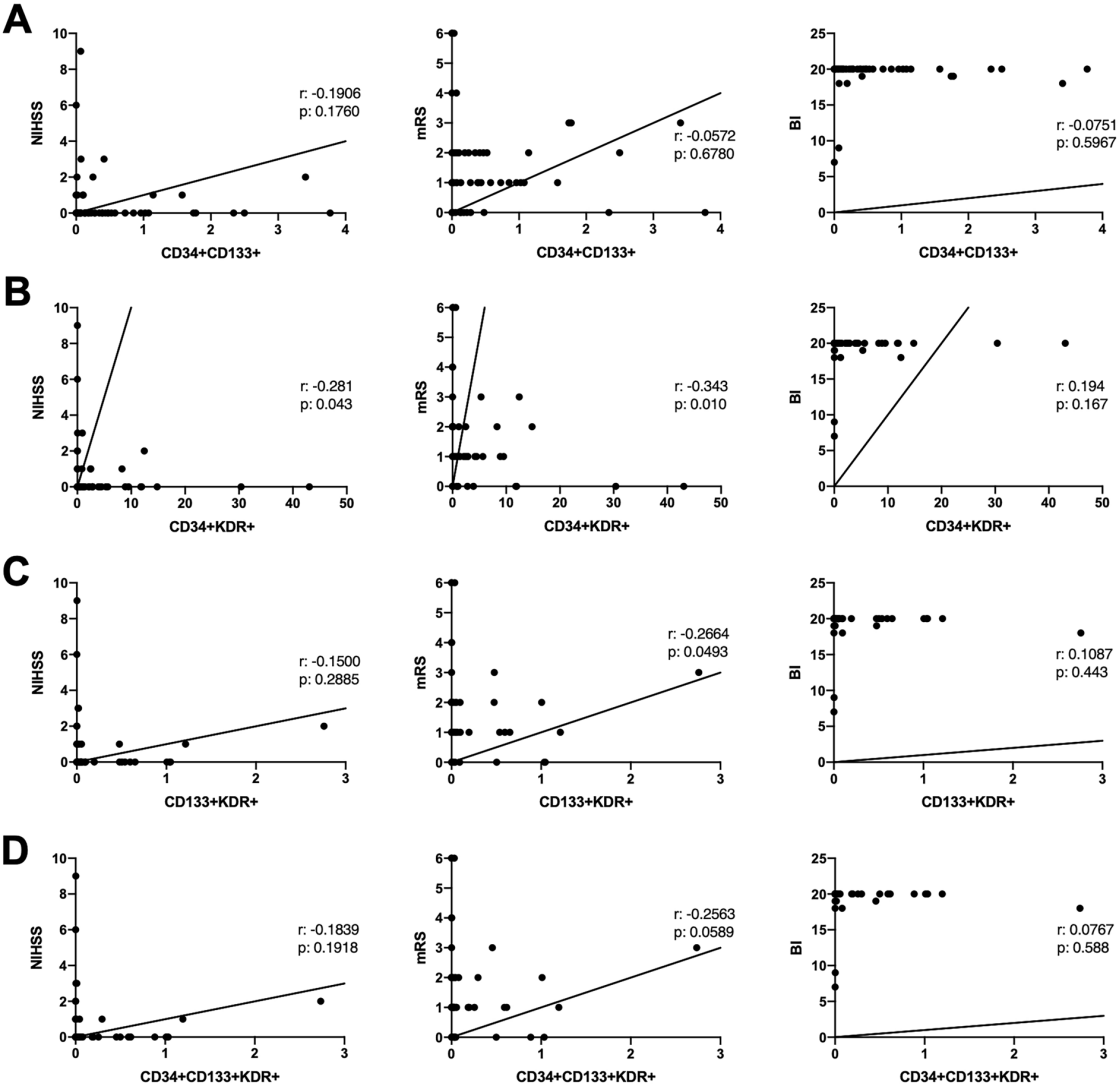
Correlation between baseline CD34 + CD133 + (**A**), CD34 + KDR + (**B**), CD133 + KDR + (**C**) and CD34 + CD133 + KDR + (**D**) counts and post-stroke severity and outcome on day 90. *NIHSS* National Institutes of Health Stroke Scale, *BI* Barthel index, *mRS* modified Rankin Score, *CD* cluster differentiation, *r* correlation coefficients, *p* p value.

Association of NIHSS, mRS and BI scores at D90 to the numbers of circulating EPC subtypes pinpointed substantial correlations between decreased BL numbers of CD34 + KDR + and stroke severity on D90 (NIHSS: r: − 0.281; *p*: 0.043; mRS: − 0.343; *p:* 0.01) (Fig. 1). In line with these findings, lower BL number of CD133 + KDR + cells were also associated with higher mRS on D90 (r: − 0.2664; *p*: 0.0493), suggesting a worse stroke outcome. Despite observation of a similar trend between BL CD34 + CD133 + KDR + numbers and D90 mRS score, the correlation remained insignificant (r: − 0.2563; *p* = 0.0589).

Similar to BL values, lower number of CD34 + KDR + cells on D7 were also associated with higher NIHSS (r: − 0.356; *p*: 0.015) and mRS (r: − 0.286; *p*: 0.049) at D90. In contrast, only reductions in D30 CD133 + KDR + (r: − 0.397; *p*: 0.006) and CD34 + CD133 + KDR + (r: − 0.399; *p*: 0.005) cell numbers were correlated with stroke severity on D90 (Supplementary Table 1). The variations in numbers of specific EPC subtypes per mL blood sample are documented in Supplementary Table 2.

### Analyses of correlation between biochemical parameters and stroke severity and outcome

**Figure 2:**
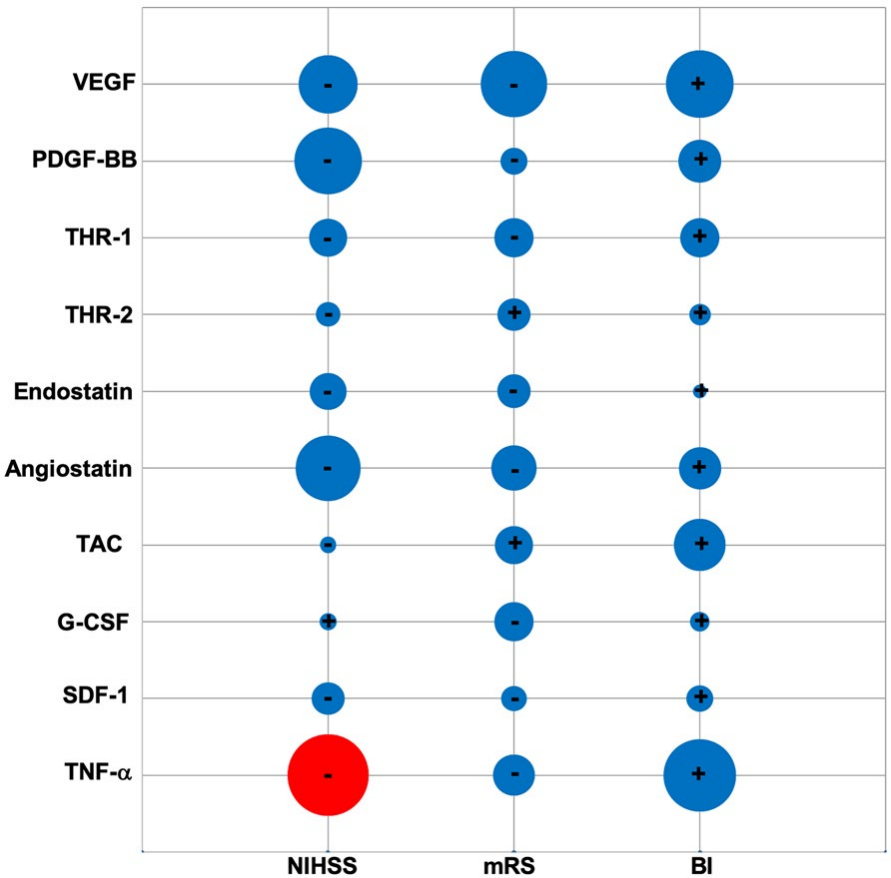
Correlation between baseline levels of angiogenic factors, total anti-oxidant capacity, chemokines and cytokines and post-stroke severity and outcome on day 90. The size of bubbles indicates the magnitude of correlation coefficient. While red and blue bubbles successively indicate significant (p < 0.05) and insignificant (p > 0.05) correlations, the symbols + and − indicate positive and negative correlations, respectively. *NIHSS* National Institutes of Health Stroke Scale, *BI* Barthel index, *mRS* modified Rankin Score, *CD* cluster differentiation, *G-CSF* granulocyte colony-stimulating factor, *PDGF-BB* platelet-derived growth factor, *SDF-1* stromal cell-derived factor-1, *TAC* total anti-oxidant capacity, *TNF-α* tumour necrosis factor-α, *THR-1* thrombospondin-1, *THR-2* thrombospondin-2, *VEGF* vascular endothelial growth factor.

Assessment of the plasmatic biochemical profile of IS patients showed no correlation between the BL levels of angiogenic modulators VEGF, PDGF-BB, thrombospondin-1/2, angiostatin and endostatin and the severity and outcome of stroke at D90 (Fig. 2). Similarly, the BL levels of TAC and plasmatic SDF-1 and G-CSF had no impact on any of the neurological and functional parameters. However, a negative correlation was detected by BL TNF-α levels and D90 NIHSS (r: − 0.343; *p*: 0.014).

**Figure 3:**
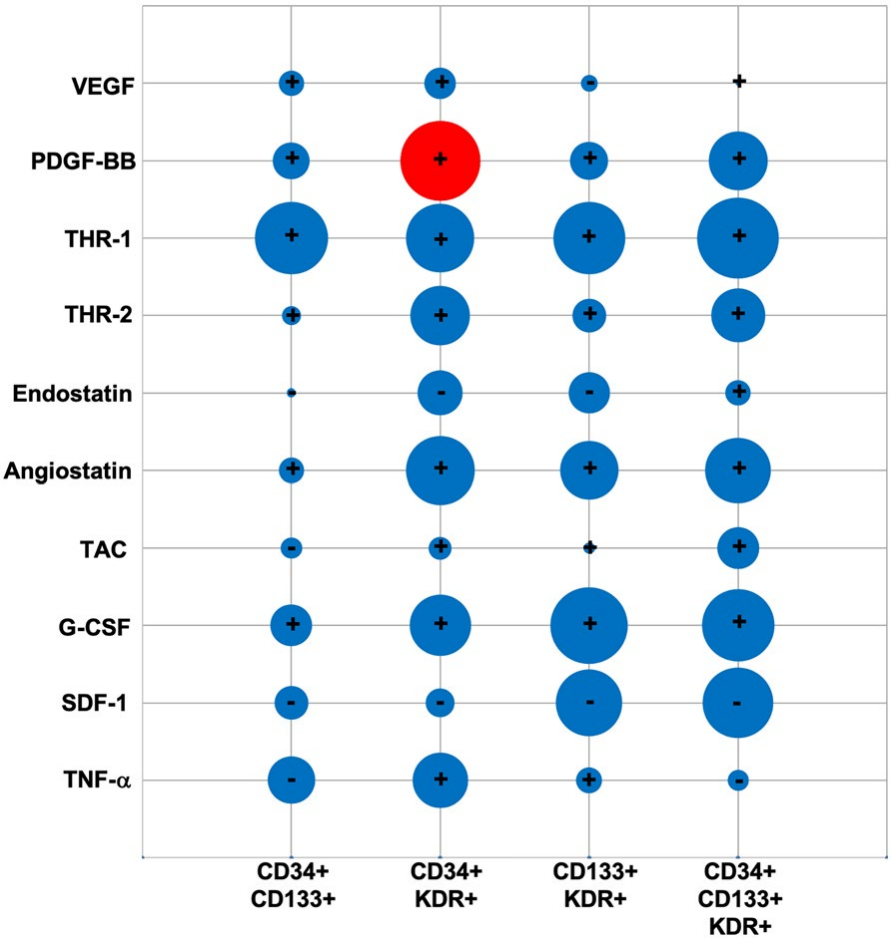
Correlation between baseline levels of angiogenic factors, total anti-oxidant capacity, chemokines and cytokines and the number of circulating EPC subtypes on day 90. The size of bubbles indicated the magnitude of correlation coefficient. While red and blue bubbles successively indicate significant (p < 0.05) and insignificant (p > 0.05) correlations, the symbols + and − indicate positive and negative correlations, respectively. *CD* cluster differentiation, *G-CSF* granulocyte colony-stimulating factor, *PDGF-BB* platelet-derived growth factor, *SDF-1* stromal cell-derived factor-1, *TAC* total anti-oxidant capacity, *TNF-α* tumour necrosis factor-α, *THR-1* thrombospondin-1, *THR-2* thrombospondin-2, *VEGF* vascular endothelial growth factor.

Scrutiny of the correlation between circulating EPC subtypes and the levels of key elements capable of affecting their counts showed that only BL level of angiogenic factor PDGF-BB (r: 0.334; *p*: 0.043) increased CD34 + KDR + numbers on D90 after stroke. In contrast, no correlation between any EPC subtype and BL levels of VEGF, thrombospondin-1/2 and endostatin were observed (Fig. 3). Again, no correlation between BL levels TAC, G-CSF, SDF-1 and TNF-α and EPC counts were observed D90 post-stroke.

Analysis of correlations between D7 and D30 levels of the same biochemical elements and the severity and outcome of stroke on D90 revealed negative correlations between D7 TAC and D90 NIHSS (r: − 0.318; *p*: 0.028) and between D30 thrombospondin-2 and D90 mRS (r: − 0.336; *p*: 0.045) scores. Interestingly, D30 thrombospondin-1 levels appeared to correlate positively with the extent of disability on D90 (r: 0.459; *p*: 0.007) as attested by the BI score (Supplementary Table 3). The actual level of biomedical parameters at BL, D7 and D30 and the neurological and functional scores on D90 are documented in the Supplementary Tables 4 and 5, respectively.

### Attempts to identify prognostic marker(s) for IS

**Figure 4:**
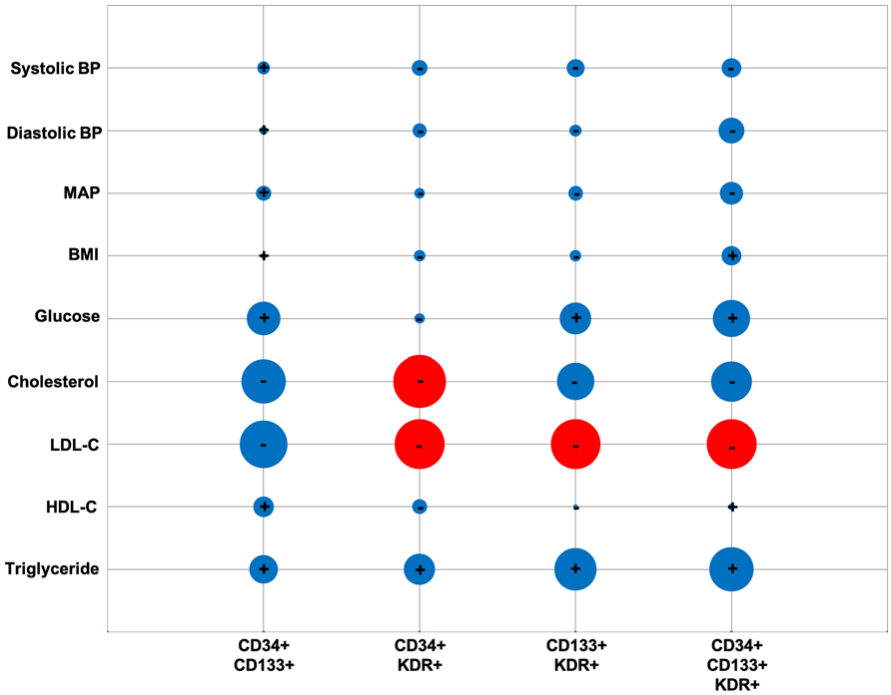
Correlation between baseline levels of biological variables, including body mass index (BMI), blood pressure (BP) and blood triglyceride, glucose and LDL- and HDL-cholesterol and the number of circulating EPC subtypes on day 90. The size of bubbles indicated the magnitude of correlation coefficient. While red and blue bubbles successively indicate significant (p < 0.05) and insignificant (p > 0.05) correlations, the symbols + and − indicate positive and negative correlations, respectively.

Assessment of biological variables, that are known to be involved in the pathogenesis as well as severity and outcome of stroke, including body mass index (BMI), systolic blood pressure and blood triglyceride, glucose and LDL-cholesterol levels, and the circulating EPC numbers showed an inverse correlation between BL cholesterol levels and D90 CD34 + KDR + numbers (r: − 0.34 *p*: 0.016). The level of LDL-cholesterol was inversely correlated with D90 numbers CD34 + KDR + (r: − 0.308; *p*: 0.035), CD133 + KDR + (r: − 0.301; *p*: 0.04) and CD34 + CD133 + KDR + (r: − 0.305; *p*: 0.037) (Fig. 4).

**Figure 5:**
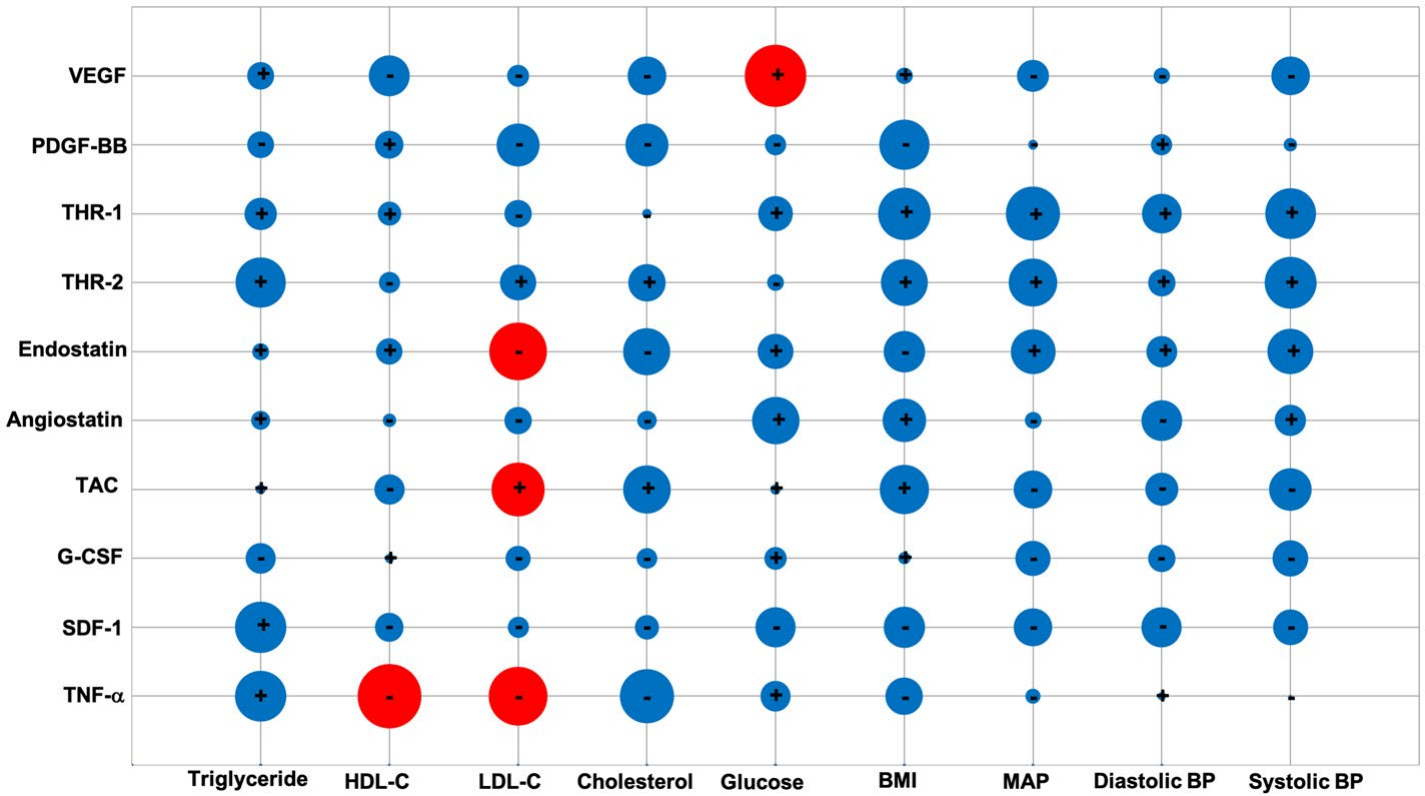
Correlation between baseline levels of biological variables, including body mass index (BMI), blood pressure (BP) and blood triglyceride, glucose and LDL- and HDL-cholesterol and the levels of angiogenic factors, total anti-oxidant capacity, cytokines and chemokines on day 90. The size of bubbles indicated the magnitude of correlation coefficient. While red and blue bubbles successively indicate significant (p < 0.05) and insignificant (p > 0.05) correlations, the symbols + and − indicate positive and negative correlations, respectively. *G-CSF* granulocyte colony-stimulating factor, *PDGF-BB* platelet-derived growth factor, *SDF-1* stromal cell-derived factor-1, *TAC* total anti-oxidant capacity, *TNF-α* tumour necrosis factor-α, *THR-1* thrombospondin-1, *THR-2* thrombospondin-2, *VEGF* vascular endothelial growth factor.

Scrutiny of the correlations between BL variables and biochemical parameters showed LDL-cholesterol were inversely correlated with D90 endostatin (r: − 0.36; *p*: 0.01) (Fig. 5). In contrast, BL glucose and LDL-C levels positively correlated with the level of VEGF (r: 0.412; *p*: 0.011) and TAC (r: 0.31; *p*: 0.036), respectively. However, LDL-C and HDL-C negatively correlated with TNF-α (r: − 0.378; *p*: 0.039; r: − 0.441; *p*: 0.012, respectively) levels on D90. No correlation was observed between any BL variable and the remaining cytokines, chemokines and angiogenesis regulators.

## Discussion

Alterations in endothelial integrity and function play a critical role in the pathogenesis of stroke and develop in otherwise healthy older individuals as part of physiological ageing process^15,16^. Besides physical damage and endothelial cell senescence, diminished availability of EPCs may also contribute to age- and stroke-mediated ED and associated vascular and functional abnormalities. Substantial decreases reported in the number of circulating EPCs expressing markers specifically for stemness and immaturity in healthy individuals over 65 years of age versus those between 18 and 64 years confirm the influence of ageing on distinct EPC subtypes^10^. In light of these and further findings implying the value of circulating EPCs for endothelial repair^6,11,17^ and as potential prognostic biomarkers^18^, the present study hypothesised that changes in circulating numbers of certain EPC subtypes, defined as cells co-expressing a variety of markers for stemness (CD34 +), immaturity (CD133 +) and endothelial cell maturity (KDR +), may explain the differences reported in the severity and/or outcome of ischaemic stroke in older patients. Observation of a significant correlation in the current study between higher CD34 + KDR + and CD133 + KDR + numbers during the acute phase of stroke and better neurological and functional outcome on D90 post-stroke corroborate this hypothesis and attribute a key role to these particular EPC subtypes in neurovascular repair^19,20^. Moreover, these findings also indicate the capacity of these particular cells to serve as prognostic markers for IS patients^21^. Since one-fourth of all mild ischaemic stroke patients manifest functional changes between days 30 and 90 after stroke, it was important to assess the level of recovery and correlate the potential changes in various biochemical compounds to recovery on D90 in this study. Indeed, neurological and functional assessments performed on D7 and D30 appear to insufficiently represent long-term recovery in mild stroke^22^.

The capacity of CD34 + KDR + cells to fully differentiate into mature endothelial cells has long been recognised^23^. Considering that the vasculoprotective effects of CD34 + cells may be explained by the paracrine effect rather than the ability of these cells to differentiate into fully functional endothelial cells, CD34 + cells continue to face scrutiny as markers of EPCs^24^. In contrast, mature endothelial cells do not express a great deal of CD133 antigens. The cells that co-express CD133 + and KDR + represent a phenotypically and functionally distinct subset of circulating endothelial cells and are widely regarded as true EPCs^25^. Observation of intracellular CD133 expression in EPCs and the diminished post-ischemic revascularisation in a nude mouse model with hind-limb ischemia following the administration of OECs transfected with specific siRNA (siCD133-EPCs) help reconcile the discrepancies regarding CD133 positivity and ontogeny in endothelial progenitors^26^.

Major growth factors such as VEGF^27^ and PDGF-BB^28^ and anti-angiogenic elements, including thrombospondin-1/2^29^, endostatin^30^ and angiostatin^31^ that regulate post-ischaemic vasculogenesis and angiogenesis may also determine the extent of neurological recovery. Unlike a recent study correlating BL plasma endostatin levels with increased risk of mortality and severe disability at 3 months, no correlation was observed between the BL plasma levels of these elements and NIHSS, mRS and BI scores at D90 in this study^32^. Although genetic variations between the two study populations, European vs Chinese, may somewhat account for the differences noted, the dichotomy in data cast doubt on the capacity of these agents to serve as highly specific and reliable prognostic markers for acute IS.

Environmental changes evoked by advanced age and ischaemic injury may suppress the production of EPCs and therefore exacerbate patients’ dependence and disability^10,11^. Oxidative stress represents one such change and evokes cerebrovascular dysfunction by not only disrupting major cellular components but also neutralising nitric oxide, a potent anti-atherogenic agent^33,34^. Albeit insignificant, the presence of a correlation between higher levels of plasma TAC and better post-stroke neurological outcome substantiates the disruptive effects of ROS on neurovasculature and pinpoints the importance of TAC in maintaining neurovascular equilibrium. Normalisation of vascular tone in dysfunctional arterial segments by ROS scavengers and antioxidant vitamins further confirm the crucial role of oxidative stress in ED^35,36^. Under normal conditions, EPCs are better protected against oxidative injury due to possession of high TAC^37,38^, but prolonged exposure to oxidative stress adversely affect their ability to proliferate, differentiate, self-renew and secrete agents with vasculoprotective capacity such as SDF-1 and VEGF^9,12,20,39^.

In addition to production, the function of EPCs may also be adversely affected by the ageing process in that suppression of systemic elements known to affect neovasculogenesis may play a crucial role^17,40^. Indeed, observation of a positive correlation between acute plasma level of PDGF-BB and CD133 + KDR +, CD34 + KDR + numbers supports this notion. The steady increases in plasma levels of PDGF-BB are likely to trigger proliferation and directed migration of EPCs to the site of injury during chronic phases of IS to counter ischaemic damage. The absence of a correlation between early SDF-1 levels and EPC counts during chronic phases of IS may be instrumental, in this context, to keep vascular growth in check^41,42^. Although SDF-1 strongly mobilizes the release of EPCs from the bone marrow^43^, the seemingly unaltered levels of SDF-1 after IS and age-mediated potential decreases in SDF-1 receptor, CXCR4 levels may explain the abovementioned lack of correlation^18^. Overall, the timing and the degree of variations observed for the parameters studied here rule out their use as reliable diagnostic markers in clinical settings.

Considering that all study participants enrolled to the study were ≥ 65 years of age, the presence of additional vascular risk factors alongside the use of certain medicines e.g. anti-platelets and anti-hypertensives may somewhat account for the differences observed in EPC counts and patients’ outcome^44,45^. Co-analyses of EPC counts with a large number of BL demographic and medical variables showed negative correlations between BL LDL-C and most EPC subtypes and specifically between BL cholesterol and CD34 + KDR + cells, confirming the point that statins significantly enhance circulating EPC numbers and pinpointing the importance of a therapy targeting cholesterol levels in stroke patients^46^. However, a recent study revealing decreases in plasma VEGF levels after statin therapy necessitate a cautious approach as regards the use of statins while signifying the importance of the type of statin used and the length of treatment received^47^. In contrast, a positive correlation between BL glucose and D90 endostatin levels indicate a prerequisite for the close monitoring of the blood glucose levels in IS patients. Positive correlations between glucose levels and endostatin levels have been established in other diseases such as diabetes and coronary artery disease^48^.

This study investigated how differences in the plasmatic availability of certain EPC subtypes during acute phase of IS might correlate with severity of disease, patients’ functional outcome and the level of agents known to affect their bioavailability. Since the numbers of patients recruited for the DMT EPC study (81 patients) and retained until D90 (62 patients) were below the originally planned number of recruits (100 patients), it is possible that the reduced sample size could have resulted in lower statistical power and might have somewhat biased the results. A bigger sample size would have been useful to better compare the differences in a large number of parameters studied. Besides, given that the majority of patients enrolled for the study had significant recovery on D90 after stroke, it is important to further investigate the correlations established in this study in patients who continue to show serious morbidity on D90.

In conclusion, circulating baseline levels of CD34 + KDR + and to a lesser degree CD133 + KDR + closely associate with the outcome of IS and may be considered as prognostic markers for IS. A future study enrolling larger numbers of patients with different degrees of morbidity is required to dis/prove this point.

### Supplementary Information

Supplementary Tables.

## Data Availability

The datasets generated during and/or analysed during the current study are available from the corresponding author on reasonable request.
