# Supplementary material for: Analysis of endothelial progenitor cell subtypes as clinical biomarkers for elderly patients with ischaemic stroke
Source: Sci Rep. 2023 Dec 9;13:21843. doi: 10.1038/s41598-023-48907-7 (PMC10710409; doi:10.1038/s41598-023-48907-7)
Supplement: Supplementary file 1 — Supplementary Tables. [file 41598_2023_48907_MOESM1_ESM.docx]

Supplementary table 1. Correlation between post-stroke days 7 and 30 EPC numbers and the severity and outcome of disease on day 90.

|  |  | Day 7 | | | Day 30 | | |
| --- | --- | --- | --- | --- | --- | --- | --- |
|  |  | **NIHSS** | **mRS** | **BI** | **NIHSS** | **mRS** | **BI** |
| CD34+CD133+ | **r** | -0.272 | 0.045 | 0.086 | -0.154 | 0.083 | -0.103 |
|  | ***p*** | 0.067 | 0.763 | 0.569 | 0.3 | 0.578 | 0.49 |
|  | ***n*** | 46 | 48 | 46 | 47 | 47 | 47 |
| CD34+KDR+ | **r** | -0.356 | -0.286 | 0.204 | -0.268 | -0.245 | 0.272 |
|  | ***p*** | **0.015** | **0.049** | 0.175 | 0.068 | 0.097 | 0.064 |
|  | ***n*** | 46 | 48 | 46 | 47 | 47 | 47 |
| CD133+KDR+ | **r** | -0.107 | -0.159 | 0.097 | -0.397 | -0.233 | 0.237 |
|  | ***p*** | 0.478 | 0.28 | 0.52 | **0.006** | 0.115 | 0.109 |
|  | ***n*** | 46 | 48 | 46 | 47 | 47 | 47 |
| CD34+CD133+  KDR+ | **r** | -0.128 | 0.013 | 0.007 | -0.399 | -0.154 | 0.267 |
|  | ***p*** | 0.395 | 0.928 | 0.965 | **0.005** | 0.301 | 0.07 |
|  | ***n*** | 46 | 48 | 46 | 47 | 47 | 47 |

NIHSS, National Institutes of Health Stroke Scale; BI, Barthel index; mRS, modified Rankin Score; CD, cluster differentiation; r, correlation coefficients; p, p value; n, number of patients.

Supplementary table 2. The mean numbers for different EPC subtypes on days 0, 7 and 30 following stroke

|  | Mean (EPC count/mL) | | |
| --- | --- | --- | --- |
|  | **Day 0** | **Day 7** | **Day 30** |
| CD34+CD133+ | 0.66 | 0.56 | 1.27 |
| CD34+KDR+ | 3.42 | 2.74 | 2.43 |
| CD133+KDR+ | 0.29 | 0.21 | 0.24 |
| CD34+CD133+KDR+ | 0.28 | 0.18 | 0.20 |

CD, cluster differentiation; EPC, endothelial progenitor cell.

Supplementary table 3. Correlation between the levels of angiogenic factors, total anti-oxidant capacity, chemokines and cytokines on post-stroke days 7 and 30 and the severity and outcome of disease on day 90.

|  |  | Day 7 | | | Day 30 | | |
| --- | --- | --- | --- | --- | --- | --- | --- |
|  |  | **NIHSS** | **mRS** | **BI** | **NIHSS** | **mRS** | **BI** |
| VEGF | **r** | -0.21 | -0.162 | 0.088 | -0.058 | -0.031 | 0.214 |
|  | ***p*** | 0.171 | 0.283 | 0.572 | 0.69 | 0.832 | 0.136 |
|  | ***n*** | 44 | 46 | 44 | 50 | 50 | 50 |
| PDGF-BB | **r** | -0.184 | -0.013 | 0.159 | -0.007 | -0.058 | 0.2 |
|  | ***p*** | 0.268 | 0.939 | 0.339 | 0.968 | 0.718 | 0.21 |
|  | ***n*** | 38 | 39 | 38 | 41 | 41 | 41 |
| THR-1 | **r** | 0.228 | 0.16 | -0.295 | -0.143 | -0.096 | 0.459 |
|  | ***p*** | 0.243 | 0.408 | 0.127 | 0.428 | 0.594 | **0.007** |
|  | ***n*** | 28 | 29 | 28 | 33 | 33 | 33 |
| THR-2 | **r** | 0.07 | -0.043 | -0.161 | -0.214 | -0.336 | 0.209 |
|  | ***p*** | 0.681 | 0.803 | 0.34 | 0.211 | **0.045** | 0.221 |
|  | ***n*** | 37 | 37 | 37 | 36 | 36 | 36 |
| Endostatin | **r** | 0.226 | 0.15 | -0.22 | -0.01 | 0.002 | 0.046 |
|  | ***p*** | 0.097 | 0.266 | 0.106 | 0.942 | 0.991 | 0.744 |
|  | ***n*** | 55 | 57 | 55 | 53 | 53 | 53 |
| Angiostatin | **r** | -0.264 | -0.049 | 0.037 | 0.072 | 0.182 | 0.021 |
|  | ***p*** | 0.096 | 0.754 | 0.821 | 0.643 | 0.237 | 0.893 |
|  | ***n*** | 41 | 43 | 41 | 44 | 44 | 44 |
| TNF-α | **r** | -0.242 | 0.15 | 0.132 | 0.214 | 0.16 | -0.104 |
|  | ***p*** | 0.143 | 0.363 | 0.431 | 0.191 | 0.331 | 0.527 |
|  | ***n*** | 38 | 39 | 38 | 39 | 39 | 39 |
| TAC | **r** | -0.318 | -0.09 | 0.064 | -0.215 | -0.252 | 0.214 |
|  | ***p*** | **0.028** | 0.541 | 0.666 | 0.134 | 0.078 | 0.136 |
|  | ***n*** | 48 | 49 | 48 | 50 | 50 | 50 |
| G-CSF | **r** | -0.252 | -0.233 | 0.132 | 0.029 | 0.004 | 0.188 |
|  | ***p*** | 0.122 | 0.148 | 0.424 | 0.867 | 0.981 | 0.265 |
|  | ***n*** | 39 | 40 | 39 | 37 | 37 | 37 |
| SDF-1 | **r** | 0.105 | 0.279 | -0.121 | -0.141 | -0.047 | 0.188 |
|  | ***p*** | 0.477 | 0.05 | 0.415 | 0.339 | 0.751 | 0.2 |
|  | ***n*** | 48 | 50 | 48 | 48 | 48 | 48 |

BI, Barthel index; CD, cluster differentiation; G-CSF, granulocyte colony-stimulating factor; mRS, modified Rankin Score; n, number of patient; NIHSS, National Institutes of Health Stroke Scale; r, correlation coefficients; p, p value; PDGF-BB, platelet-derived growth factor; SDF-1, stromal cell-derived factor-1; TAC, total anti-oxidant capacity; TNF-α, tumour necrosis factor-α; THR-1, thrombospondin-1; THR-2, thrombospondin-2; VEGF, vascular endothelial growth factor.

Supplementary table 4. The mean values for the levels of angiogenic factors, total anti-oxidant capacity, chemokines and cytokines on days 0, 7 and 30 following stroke

|  | Day 0 | Day 7 | Day 30 |
| --- | --- | --- | --- |
| VEGF (pg/mL) | 108.54 | 87.58 | 76.1 |
| TNF-α (pg/mL) | 1.41 | 1.53 | 1.60 |
| THR-1 (ng/mL) | 2085.50 | 1359.59 | 1220.74 |
| THR-2 (ng/mL) | 27.78 | 24.011 | 34.59 |
| TAC (TEC/nM) | 16.19 | 14.97 | 14.03 |
| PDGF-BB (pg/mL) | 326.35 | 245.44 | 325.35 |
| G-CSF (pg/mL) | 31.19 | 36.83 | 34.91 |
| Endostatin (ng/mL) | 278.10 | 222.88 | 313.76 |
| SDF-1 (pg/mL) | 2334.11 | 2393.23 | 2316.34 |
| Angiostatin (ng/mL) | 258.09 | 308.30 | 291.38 |

G-CSF, granulocyte colony-stimulating factor; PDGF-BB, platelet-derived growth factor; SDF-1, stromal cell-derived factor-1; TAC, total anti-oxidant capacity; TEC, Trolox equivalent capacity; TNF-α, tumour necrosis factor-α; THR-1, thrombospondin-1; THR-2, thrombospondin-2; VEGF, vascular endothelial growth factor.

Supplementary table 5. The mean values for NIHSS, BI and mRS scores on day 90 after stroke

|  | Mean |
| --- | --- |
| NIHSS | 4.04 |
| mRS | 2.05 |
| BI | 19.6 |

BI, Barthel index; mRS, modified Rankin Score; NIHSS, National Institutes of Health Stroke Scale.
